# Explicit maps to predict activation order in multiphase rhythms of a coupled cell network

**DOI:** 10.1186/2190-8567-2-4

**Published:** 2012-03-12

**Authors:** Jonathan E Rubin, David Terman

**Affiliations:** 1 Department of Mathematics, University of Pittsburgh, Pittsburgh, PA, 15260, USA; 2 Department of Mathematics, The Ohio State University, Columbus, OH, 43210, USA

**Keywords:** fast-slow analysis, clustered solutions, map, multiphase rhythm, respiration

## Abstract

We present a novel extension of fast-slow analysis of clustered solutions to coupled networks of three cells, allowing for heterogeneity in the cells’ intrinsic dynamics. In the model on which we focus, each cell is described by a pair of first-order differential equations, which are based on recent reduced neuronal network models for respiratory rhythmogenesis. Within each pair of equations, one dependent variable evolves on a fast time scale and one on a slow scale. The cells are coupled with inhibitory synapses that turn on and off on the fast time scale. In this context, we analyze solutions in which cells take turns activating, allowing any activation order, including multiple activations of two of the cells between successive activations of the third. Our analysis proceeds via the derivation of a set of explicit maps between the pairs of slow variables corresponding to the non-active cells on each cycle. We show how these maps can be used to determine the order in which cells will activate for a given initial condition and how evaluation of these maps on a few key curves in their domains can be used to constrain the possible activation orders that will be observed in network solutions. Moreover, under a small set of additional simplifying assumptions, we collapse the collection of maps into a single 2D map that can be computed explicitly. From this unified map, we analytically obtain boundary curves between all regions of initial conditions producing different activation patterns.

## 1 Introduction

 The methods of fast-slow decomposition have been harnessed for the analysis of rhythmic activity patterns in many mathematical models of single excitable or oscillatory elements featuring two or more time scales. In the analysis of relaxation oscillations, for example, singular solutions can be formed by concatenating slow trajectories associated with silent and active phases and fast jumps between these phases, and these can guide the study of true solutions. These methods can be productively extended to interacting pairs of elements, particularly when the coupling between them takes certain forms. The synaptic coupling that arises in many neuronal contexts is well suited for the use of this theory. In the case of synapses that turn on and off on the fast time scale, for example, analysis can be performed through the use of separate phase spaces for each neuron, with synaptic inputs modifying the nullsurfaces and other relevant structures in each phase space. This method has been used to treat pairs of neurons with slow synaptic dynamics as well, although higher-dimensional phase spaces arise. Similarly, synchronized and clustered solutions can be analyzed in model networks consisting of multiple identical neurons if these neurons are visualized as multiple particles in one phase space or in two phase spaces, one for active neurons and one for silent, the membership of which will change over time. Reviews of how fast-slow decompositions have been used to analyze neuronal networks can be found in, for example, [1,2]. 

This form of analysis becomes significantly more challenging when networks of three or more nonidentical neurons are considered. The number of variables in each slow subsystem can become prohibitive, and if variables associated with different neurons are considered in separate phase spaces, then some method is still needed for the efficient analysis of their interactions. In this study, we introduce such a method, based on mappings on slow variables, for networks in which each element is modeled with one fast variable and one slow variable, plus a coupling variable. A strength of this method is that, by numerically computing the locations of a few key curves in phase space, we can obtain information about model trajectories generated by arbitrary initial conditions and determine how complex changes in stable firing patterns occur as parameters are varied. Moreover, the formulas defining approximations to these curves, valid under a small number of simplifying assumptions, can be expressed in an elegant analytical form. These methods are particularly tractable within networks consisting of three reciprocally coupled units, so we focus on such networks here; also, we use intrinsic dynamics arising in neuronal models, although the theory would work identically for any qualitatively similar dynamics with two time scales.

 Although three-component models arise in many applications, in neuroscience and beyond, our original motivation for this work comes from the study of networks in the mammalian brain stem that generate respiratory rhythms [3]. A brief description of modeling work related to these rhythms is given in the following section. This description is followed by the equations for a particular reduced model for the respiratory network that we consider. In Section 3, we present examples of complex firing patterns that arise as solutions to the model to motivate the analysis that follows. We next demonstrate how fast-slow analysis can be used to derive reduced equations for the evolution of solutions during both the silent and active phases. In particular, we derive formulas for the times when each cell jumps up and down, and determine how these times depend on parameters and initial conditions. To derive these explicit formulas, we will make some simplifying assumptions on the equations; a similar analysis could be performed numerically if such explicit formulas could not be obtained. In Section 4, we make some further simplifying assumptions that allow us to reduce the full dynamics to a piecewise continuous two-dimensional map. Analysis of this map helps to explain how complex transitions in stable firing patterns take place as parameters are varied. We conclude the article with a discussion in Section 5.

## 2 Model system

### 2.1 Modeling respiratory rhythms

 Recent work, based on experimental observations, has modeled the respiratory rhythm generating network in the brain stem as a collection of four or five neuronal populations. Three of these groups are inhibitory and are arranged in a ring, with each population inhibiting the other two. A fourth group, a relatively well-studied collection of neurons in the pre-Bötzinger Complex (pre-BötC), excites one of the inhibitory populations, also associated with the pre-BötC, and is inhibited by the other two. Finally, some studies have included a fifth, excitatory population, linked to certain other populations and likely becoming active only under certain strong perturbations to environmental or metabolic conditions [4-8]. In addition to the synaptic inputs from other populations in the network, each neuronal group receives excitatory synaptic drives from one or more additional sources, possibly related to feedback control of respiration (e.g., [9]). Under baseline conditions, the four core populations encompassed in this model generate a rhythmic output, in which the inhibitory groups take turns firing and the activity of the excitatory pre-BötC neurons slightly leads but largely overlaps that of the inhibitory pre-BötC cells. 

 In some of this work, a model respiratory network in which each population consists of a heterogeneous collection of fifty Hodgkin-Huxley neurons was constructed and tuned to reproduce a range of experimental observations in simulations [4,5,7]. Achieving this data fitting presumably required a major effort to select values for the many unknown parameters in the model. A reduced version of this model network, in which each population was modeled by a single coupled pair of ordinary differential equations, was also developed and, after parameter tuning, some analysis was performed to describe its activity in terms of fast and slow dynamics and transitions by escape and release [6,8]. Although the reduced population model involves far fewer free parameters than the Hodgkin-Huxley type model, it still includes coupling strengths between all the synaptically connected populations, drive strengths, and adaptation time scales, among others, amounting collectively to a many-dimensional parameter space. Thus, selecting parameter values for which model behavior matches experimental findings and determining which parameter values produce what forms of dynamics represent burdensome numerical tasks. These challenges are significantly complicated by the possibility of multistability, as different initial conditions could lead to different solutions for each parameter set. 

 The method that we present in this study has been developed to aid in the analytical study of solutions of networks like the reduced respiratory population model. To make the presentation concrete, we present our results in terms of this model. Since two of the four active populations relevant to the normal respiratory rhythm, those in the pre-BötC, activate in near-synchrony, we will treat these as a single population and consider a three population network. The activity of one of the key respiratory brain stem populations depends on a persistent sodium current [10-13], while the other active populations feature an adaptation current instead [5,6]. In the three population model that we use, we include this heterogeneity to illustrate that the theory handles heterogeneity easily, to distinguish one of the populations from the other two for ease of presentation of part of the theory, and to maintain a strong connection with the respiratory application. 

### 2.2 The equations

The model equations we consider are

(1) $$\begin{array}{rcl} v_{1}^{\prime } & = & F_{1}(v_{1},h)-g_{I}\left(b_{21}S_{\infty }(v_{2})+b_{31}S_{\infty }(v_{3})\right)(v_{1}-V_{I})-g_{E}d_{1}(v_{1}-V_{E}),\\ v_{2}^{\prime } & = & F_{2}(v_{2},m_{2})-g_{I}\left(b_{12}S_{\infty }(v_{1})+b_{32}S_{\infty }(v_{3})\right)(v_{2}-V_{I})-g_{E}d_{2}(v_{2}-V_{E}),\\ v_{3}^{\prime } & = & F_{3}(v_{3},m_{3})-g_{I}\left(b_{13}S_{\infty }(v_{1})+b_{23}S_{\infty }(v_{2})\right)(v_{3}-V_{I})-g_{E}d_{3}(v_{3}-V_{E}),\\ h^{\prime } & = & \epsilon \left(h_{\infty }(v_{1})-h\right)/\tau_{h}(v_{1}),\\ m_{2}^{\prime } & = & \epsilon \left(m_{\infty }(v_{2})-m_{2}\right)/\tau_{2}(v_{2}),\\ m_{3}^{\prime } & = & \epsilon \left(m_{\infty }(v_{3})-m_{3}\right)/\tau_{3}(v_{3}). \end{array}$$

Differentiation is with respect to time *t*, and *ϵ* is a small, positive parameter that we have introduced for notational convenience. In [6,8], each *v* variable denotes the average voltage over a synchronized neuronal population, *h* is the inactivation of a persistent sodium current for members of the inspiratory pre-BötC population, and the $m_{i}$ represent the activation levels of an adaptation current for two other respiratory populations; however, each variable could just as easily represent analogous quantities for a single neuron.

The functions $F_{i}$ in (1) are given by:

(2) $$\begin{array}{rcl} F_{1}(v_{1},h) & = & -\left(I_{NaP}(v_{1},h)+I_{Kdr}(v_{1})+I_{L}(v_{1})\right)/C,\\ F_{2}(v_{2},m_{2}) & = & -\left(I_{ad}(v_{2},m_{2})+I_{L}(v_{2})\right)/C,\\ F_{3}(v_{3},m_{3}) & = & -\left(I_{ad}(v_{3},m_{3})+I_{L}(v_{3})\right)/C, \end{array}$$

where *C* is membrane capacitance and $I_{NaP}(v,h)=g_{NaP}m_{p_{\infty }}(v)h(v-V_{Na})$, $I_{Kdr}(v)=g_{Kdr}n_{\infty }^{4}(v)(v-V_{K})$, $I_{L}(v)=g_{L}(v-V_{L})$, and $I_{ad}(v,m)=g_{ad}m(v-V_{K})$ represent persistent sodium, potassium, leak and adaptation currents, respectively. In each of these currents, the *g* parameter denotes conductance and the *V* parameter is the current’s reversal potential. We use the standard convention of representing $I_{NaP}$ and $I_{Kdr}$ activation as sigmoidal functions of voltage *v*, $m_{p_{\infty }}(v)$ and $n_{\infty }(v)$, respectively. The coupling function in system (1) is given by $S_{\infty }(v)=1//\{1+\exp[(v-\theta_{I})/\sigma_{I}]\}$, which closely approximates a Heaviside step function due to the small size of $\sigma_{I}$ and which is multiplied by a strength factor *b* each time it appears. The final term, $g_{E}d_{i}(v_{i}-V_{E})$, in each voltage equation represents a tonic synaptic drive from a feedback population; the strength factors $d_{i}$ could change with changing metabolic or environmental conditions, but we treat them as constants in this article. Additional details about the functions in (1) and (2), as well as parameter values used, are given in Appendix 1. Appendix 2 also presents a general list of assumptions, satisfied by (1), (2) with the parameter values used, under which our theoretical methods will work.

## 3 Fast-slow analysis

### 3.1 Introduction

**Fig. 1:**
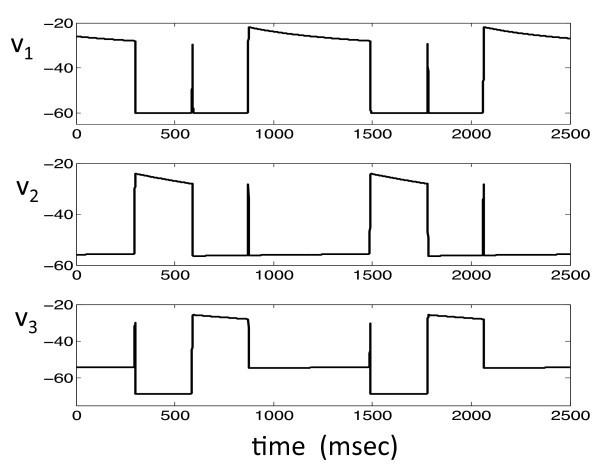
A typical solution of system (1). There is always one and only one cell active at each time. When an active cell’s voltage reaches the synaptic threshold $\theta_{I}$, it jumps down releasing the other two cells from inhibition. There is then a race among these two cells to see which one crosses the synaptic threshold first. The winning cell becomes active and the other two cells return to the silent phase.

**Fig. 2:**
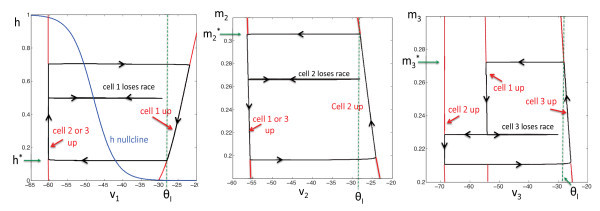
The projections of the solution shown in Figure 1 onto the phase planes corresponding to the three cells. A cell lies on the left branch of its *v*-nullcline while in the silent phase and on the right branch during the active phase. Jumps up and down between these branches are initiated when an active cell reaches the synaptic threshold $\theta_{I}$, which occurs at $h=h^{*}$, $m_{2}=m_{2}^{*}$, or $m_{3}=m_{3}^{*}$, respectively.

A typical solution of system (1) is shown in Figure 1. Each of the cells lies in one of four states, which we denote as: (i) the silent phase; (ii) the active phase; (iii) the jump-up; and (iv) the jump-down. For example, in Figure 1, at t=0, cell 1 is active, while cells 2 and 3 are silent. At this time, cell 1 inhibits both of the other cells. This configuration is maintained until $v_{1}(t)$ crosses the synaptic threshold $\theta_{I}$, at which point the inhibitory input to cells 2 and 3 is turned off. Both cells 2 and 3 will then begin to jump up to the active phase (due to post-inhibitory rebound, which will be discussed shortly). There is then a race to see which of the voltages, $v_{2}(t)$ or $v_{3}(t)$, crosses the threshold $\theta_{I}$ first. Suppose that $v_{2}$ crosses $\theta_{I}$ first, as in the first transition that occurs in Figure 1. When this happens, cell 2 sends inhibition to both cells 1 and 3, so both of these cells must return to the silent phase. Hence, cell 2 is now active, while the other two cells are silent. These roles persist until $v_{2}(t)$ crosses the synaptic threshold $\theta_{I}$ and releases cells 1 and 3 from inhibition, at which time there is another race to see whether cell 1 or cell 3 crosses threshold first. This process continues, with one of the cells always lying in the active phase until its membrane potential crosses threshold and releases the other two cells from inhibition. The projections of this solution onto the phase planes corresponding to the three cells are shown in Figure 2. 

We analyze solutions using fast-slow analysis. The basic idea is that the solution evolves on two different time scales: During the jumps up and down, the solution evolves on a fast time scale, while during the silent and active phases, the solution evolves on a slow time scale. The fast-slow analysis allows us to derive reduced equations that determine the evolution of the solution during each of these phases. In particular, we derive explicit formulas for the times when each cell jumps up and down and use these to determine the outcomes of the races to threshold, depending on parameters and initial conditions. To derive these formulas, we will make some simplifying assumptions on the equations; in situations in which such formulas cannot be obtained, then a similar analysis can be done numerically.

### 3.2 Slow and fast equations

We first consider equations for the slow variables *h*, $m_{1}$ and $m_{2}$. These equations are obtained by introducing the slow time scale, τ=ϵt, and then setting ϵ=0 in the resulting equations. These steps give:

(3) $$\begin{array}{rcl} 0 & = & F_{1}(v_{1},h)-g_{I}\left(b_{21}S_{\infty }(v_{2})+b_{31}S_{\infty }(v_{3})\right)(v_{1}-V_{I})-g_{E}d_{1}(v_{1}-V_{E}),\\ 0 & = & F_{2}(v_{2},m_{2})-g_{I}\left(b_{12}S_{\infty }(v_{1})+b_{32}S_{\infty }(v_{3})\right)(v_{2}-V_{I})-g_{E}d_{2}(v_{2}-V_{E}),\\ 0 & = & F_{3}(v_{3},m_{3})-g_{I}\left(b_{13}S_{\infty }(v_{1})+b_{23}S_{\infty }(v_{2})\right)(v_{3}-V_{I})-g_{E}d_{3}(v_{3}-V_{E}),\\ h^{\prime } & = & \left(h_{\infty }(v_{1})-h\right)/\tau_{h}(v_{1}),\\ m_{2}^{\prime } & = & \left(m_{\infty }(v_{2})-m_{2}\right)/\tau_{2}(v_{2}),\\ m_{3}^{\prime } & = & \left(m_{\infty }(v_{3})-m_{3}\right)/\tau_{3}(v_{3}), \end{array}$$

where differentiation is with respect to *τ*. To simplify the analysis, we take the extreme (v→±∞) values of each of the functions $h_{\infty }$, $m_{\infty }$, $\tau_{h}$, $\tau_{2}$, and $\tau_{3}$ and replace each function with a step function that jumps abruptly between these values. That is, we assume that there are positive constants $\sigma_{L}$, $\sigma_{R}$, $\lambda_{L}$, $\lambda_{R}$, $\mu_{L}$ and $\mu_{R}$ (see Tables 1 and 2 in Appendix 1, singular limit parameter values) such that the slow variables satisfy equations of the form:

$$\begin{array}{rcl} h^{\prime }(t) & = & \left\{ \begin{array}{ll} \sigma_{L}(1-h) & \text{if cell 1 is silent},\\ -\sigma_{R}h & \text{if cell 1 is active}, \end{array}\right.\\ m_{2}^{\prime }(t) & = & \left\{ \begin{array}{ll} -\lambda_{L}m_{2} & \text{if cell 2 is silent},\\ \lambda_{R}(1-m_{2}) & \text{if cell 2 is active}, \end{array}\right.\\ m_{3}^{\prime }(t) & = & \left\{ \begin{array}{ll} -\mu_{L}m_{3} & \text{if cell 3 is silent},\\ \mu_{R}(1-m_{3}) & \text{if cell 3 is active}. \end{array}\right. \end{array}$$

We solve these equations explicitly to obtain:

(4) $$h(\tau )=\left\{ \begin{array}{ll} 1+\left(h(0)-1\right)e^{-\sigma_{L}\tau } & \text{if cell 1 is silent},\\ h(0)e^{-\sigma_{R}\tau } & \text{if cell 1 is active}, \end{array}\right.$$

(5) $$m_{2}(\tau )=\left\{ \begin{array}{ll} m_{2}(0)e^{-\lambda_{L}\tau } & \text{if cell 2 is silent},\\ 1+\left(m_{2}(0)-1\right)e^{-\lambda_{R}\tau } & \text{if cell 2 is active}, \end{array}\right.$$

(6) $$m_{3}(\tau )=\left\{ \begin{array}{ll} m_{3}(0)e^{-\mu_{L}\tau } & \text{if cell 3 is silent},\\ 1+\left(m_{3}(0)-1\right)e^{-\mu_{R}\tau } & \text{if cell 3 is active}. \end{array}\right.$$

**Table 1:** Parameter values for full model and singular limit simulations and singular limit analysis corresponding to Figure 3

Conductances (nS)	Reversal potentials (mV)	Half activations (mV)	Slopes	Time constants (ms)	Coupling constants	Other
$g_{NaP}=0.25$	$V_{Na}=50$	$\theta_{h}=-48$	$\sigma_{h}=3$	$\tau_{a,h}=9.5$	$b_{12}=0.4$	*ϵ* = 0.01
$g_{Kdr}=0.25$	$V_{K}=-85$	$\theta_{n}=-30$	$\sigma_{n}=-4$	$\tau_{a,2}=30$	$b_{13}=0.4$	C=1 pF
$g_{ad}=0.5$		$\theta_{m}=-36$	$\sigma_{m}=-10^{-1}$	$\tau_{a,3}=45$	$b_{21}=0.2$	$d_{1}=0.21$
$g_{L}=0.14$	$V_{L}=-60$	$\theta_{m_{p}}=-50$	$\sigma_{m_{p}}=-10^{-1}$	$\tau_{b,h}=-4.5$	$b_{23}=0.24$	$d_{2}=0.73$
$g_{I}=3.0$	$V_{I}=-75$	$\theta_{h}^{\tau }=-48$	$\sigma_{h}^{\tau }=-10^{-2}$	$\tau_{b,2}=-10$	$b_{31}=0.3$	$d_{3}=1.4$
$g_{E}=0.5$	$V_{E}=0$	$\theta_{2}^{\tau }=0$	$\sigma_{2}^{\tau }=10^{-1}$	$\tau_{b,3}=-32.3$	$b_{32}=0.25$	$\theta_{I}=-32$
		$\theta_{3}^{\tau }=0$	$\sigma_{3}^{\tau }=10^{-1}$			$\sigma_{I}=-10^{-1}$
Singular limit parameter values	$\sigma_{L}=\frac{1}{950}$	$\sigma_{R}=\frac{1}{500}$	$\lambda_{L}=\frac{1}{2\text{,}000}$	$\lambda_{R}=\frac{1}{2\text{,}000}$	$\mu_{L}=\frac{1}{1\text{,}270}$	$\mu_{R}=\frac{1}{1\text{,}270}$

**Table 2:** Parameter values for full model and singular limit simulations and singular limit analysis corresponding to Figures 5A and 6A

Conductances (nS)	Reversal potentials (mV)	Half activations (mV)	Slopes	Time constants (ms)	Coupling constants	Other
$g_{NaP}=0.25$	$V_{Na}=50$	$\theta_{h}=-48$	$\sigma_{h}=3$	$\tau_{a,h}=5$	$b_{12}=0.4$	*ϵ* = 0.01
$g_{Kdr}=0.25$	$V_{K}=-85$	$\theta_{n}=-30$	$\sigma_{n}=-4$	$\tau_{a,2}=35$	$b_{13}=0.5$	C=1 pF
$g_{ad}=0.6$		$\theta_{m}=-40$	$\sigma_{m}=-10^{-3}$	$\tau_{a,3}=20$	$b_{21}=0.3$	$d_{1}=0.55$
$g_{L}=0.2$	$V_{L}=-60$	$\theta_{m_{p}}=-40$	$\sigma_{m_{p}}=-10^{-2}$	$\tau_{b,h}=-1.5$	$b_{23}=0.5$	$d_{2}=1.4$
$g_{I}=3.0$	$V_{I}=-75$	$\theta_{h}^{\tau }=-48$	$\sigma_{h}^{\tau }=-10^{-2}$	$\tau_{b,2}=0$	$b_{31}=0.3$	$d_{3}=1.5$
$g_{E}=0.4$	$V_{E}=0$	$\theta_{2}^{\tau }=-40$	$\sigma_{2}^{\tau }=10^{-3}$	$\tau_{b,3}=0$	$b_{32}=2.0$	$\theta_{I}=-40$
		$\theta_{3}^{\tau }=-40$	$\sigma_{3}^{\tau }=10^{-3}$			$\sigma_{I}=-10^{-3}$
Singular limit parameter values	$\sigma_{L}=\frac{1}{500}$	$\sigma_{R}=\frac{1}{350}$	$\lambda_{L}$: see text	$\lambda_{R}=\frac{1}{3\text{,}500}$	$\mu_{L}$: see text	$\mu_{R}=\frac{1}{2\text{,}000}$

We next consider the fast time scale, which is simply *t*. Let ϵ=0 in (1) to obtain the fast equations:

(7) $$\begin{array}{rcl} v_{1}^{\prime } & = & F_{1}(v_{1},h)-g_{I}\left(b_{21}S_{\infty }(v_{2})+b_{31}S_{\infty }(v_{3})\right)(v_{1}-V_{I})-g_{E}d_{1}(v_{1}-V_{E}),\\ v_{2}^{\prime } & = & F_{2}(v_{2},m_{2})-g_{I}\left(b_{12}S_{\infty }(v_{1})+b_{32}S_{\infty }(v_{3})\right)(v_{2}-V_{I})-g_{E}d_{2}(v_{2}-V_{E}),\\ v_{3}^{\prime } & = & F_{3}(v_{3},m_{3})-g_{I}\left(b_{13}S_{\infty }(v_{1})+b_{23}S_{\infty }(v_{2})\right)(v_{3}-V_{I})-g_{E}d_{3}(v_{3}-V_{E}),\\ h^{\prime } & = & m_{2}^{\prime }=m_{3}^{\prime }=0. \end{array}$$

Note that the slow variables are constant on the fast time scale. We will only explicitly solve the fast equations when there is no inhibition; that is, we will solve these equations to determine what happens when the cells are released from inhibition (which we take to be at t=0) and jump up, competing to become active next. In this case, each $S_{\infty }=0$. We note that the fast equations for $v_{2}$ and $v_{3}$ are both linear and can be solved explicitly. If there is no inhibitory input then, for k=2 or 3,

(8) $$v_{k}(t)=A_{k}+\left(v_{k}(0)-A_{k}\right)e^{-B_{k}t}.$$

Since $V_{E}=0$ (see Tables 1 and 2 in Appendix 1), this gives

$$A_{k}=\frac{g_{ad}m_{k}V_{K}+g_{L}V_{L}}{g_{ad}m_{k}+g_{L}+g_{E}d_{k}}\;\text{and}\;B_{k}=g_{ad}m_{k}+g_{L}+g_{E}d_{k}.$$

To obtain an explicit formula for $v_{1}(t)$, we will make some simplifying assumptions. First, since the voltage values for cell 1 during the silent phase and most of the jump up lie in a range where the potassium activation function $n_{\infty }(v)$ is quite small, we assume that $n_{\infty }(v_{1})$ is negligible throughout these phases. Moreover, we assume that the sodium gating variable $m_{p_{\infty }}(v)$ is a step function. That is, there is a threshold value, $V_{mp}<\theta_{I}$, so that $m_{p_{\infty }}(v)=0$ if $v<V_{mp}$ and $m_{p_{\infty }}(v)=1$ if $v>V_{mp}$. In this case, the fast equation for $v_{1}$ is piecewise linear, and we can write its solution as

(9) $$v_{1}(t)=\{\begin{array}{cc} A_{1}+\left(v_{1}(0)-A_{1}\right)e^{-B_{1}t}, & 0\leq t<t_{mp},\\ {\hat{A}}_{1}+(V_{mp}-{\hat{A}}_{1})e^{-{\hat{B}}_{1}(t-t_{mp})}, & t_{mp}\leq t, \end{array}$$

where

$$\begin{array}{rcl} A_{1} & = & g_{L}V_{L}/B_{1},\;B_{1}=g_{L}+g_{E}d_{1},\\ {\hat{A}}_{1} & = & (g_{NaP}hV_{Na}+g_{L}V_{L})/{\hat{B}}_{1},\;\text{and}\;{\hat{B}}_{1}=g_{NaP}h+g_{L}+g_{E}d_{1}, \end{array}$$

with

$$t_{mp}=\frac{1}{B_{1}}\ln\frac{B_{1}v_{1}(0)-g_{L}V_{L}}{B_{1}V_{mp}-g_{L}V_{L}}.$$

### 3.3 The race

As described above, when one of the cells jumps down, there is a race to see which of the other cells reaches threshold first and then inhibits the other cells. Here we derive formulas that determine which cell wins the race to threshold.

First suppose that cell 1 jumps down from the active phase and releases cells 2 and 3 from inhibition. We need to determine the times it takes for the membrane potentials of these two cells to reach the synaptic threshold. While jumping up, these membrane potentials satisfy (8), so once we determine the initial conditions $v_{k}(0)$, k=2,3, we can solve for the jump-up times.

While cells 2 and 3 are in the silent phase, they lie on the slow nullclines given by the second and third equations in (3) with $S_{\infty }(v_{1})=1$ and $S_{\infty }(v_{2})=S_{\infty }(v_{3})=0$. Given any values of $m_{2}$ and $m_{3}$, we can solve these equations explicitly for $v_{2}$ and $v_{3}$ to conclude that at the moment that cells 2 and 3 begin to jump up,

(10) $$v_{k}(0)=\frac{g_{ad}m_{k}V_{K}+g_{L}V_{L}+g_{I}b_{1k}V_{I}}{g_{ad}m_{k}+g_{L}+g_{I}b_{1k}+g_{E}d_{k}}\equiv V_{k1},$$

where k=2 or 3. Substituting this expression into (8) and setting $v_{k}(t_{k1})=\theta_{I}$, we find that the jump-up times are given by

(11) $$t_{k1}(m_{k})=\frac{1}{C_{k1}}\ln\left(\frac{D_{k1}}{E_{k1}}\right),$$

where

$$\begin{array}{rcl} C_{k1} & = & g_{ad}m_{k}+g_{L}+g_{E}d_{k},\\ D_{k1} & = & g_{ad}m_{k}(V_{k1}-V_{K})+g_{L}(V_{k1}-V_{L})+d_{k}g_{E}V_{k1} \end{array}$$

and

$$E_{k1}=g_{ad}m_{k}(\theta_{I}-V_{K})+g_{L}(\theta_{I}-V_{L})+d_{k}g_{E}\theta_{I}.$$

**Fig. 3:**
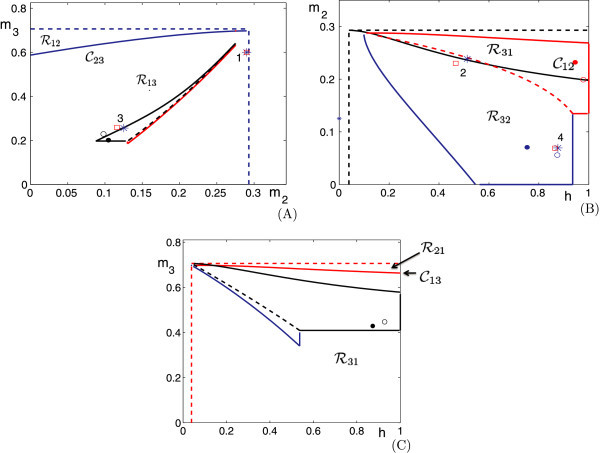
Jumping regions in the slow phase planes. **(A)**$\left(m_{2},m_{3}\right)$ plane. **(B)**$\left(h,m_{2}\right)$ plane. **(C)**$\left(h,m_{3}\right)$ plane. Curves and color codes are described in detail in the text.

Now, either cell 2 or cell 3 will win the race, if either $t_{21}(m_{2})<t_{31}(m_{3})$ or $t_{21}(m_{2})>t_{31}(m_{3})$, respectively. The equation $t_{21}(m_{2})=t_{31}(m_{3})$ defines a curve in the $\left(m_{2},m_{3}\right)$ plane, which we denote as $C_{23}$. An example of this curve is shown in Figure 3A, where we numerically solved for $C_{23}$ for parameter values given in Table 1 in the Appendix. Points above this curve correspond to cell 2 winning the race and points below this curve correspond to cell 3 winning the race. 

Next suppose that cell *k*, k=2 or 3, wins the race. When cell *k* jumps down from the active phase, cells 1 and *j*, j=2 or 3 and j≠k, are released from inhibition. We repeat our calculation for the race that ensues. Specifically, we obtain the initial condition $v_{j}(0)$ from Equation (10) and compute $v_{1}(0)$ analogously, by considering the first equation in (3) with $S_{\infty }(v_{k})=1$, $S_{\infty }(v_{1})=S_{\infty }(v_{j})=0$ and $m_{p_{\infty }}(v_{1})=n_{\infty }(v_{1})=0$. These steps give

(12) $$v_{j}(0)=\frac{g_{ad}m_{j}V_{K}+g_{L}V_{L}+g_{I}b_{kj}V_{I}}{g_{ad}m_{j}+g_{L}+g_{I}b_{kj}+g_{E}d_{j}}\equiv V_{jk},$$

(13) $$v_{1}(0)=\frac{g_{L}V_{L}+g_{I}b_{k1}V_{I}}{g_{L}+g_{I}b_{k1}+g_{E}d_{1}}\equiv V_{1k}.$$

As with the derivation of (11), substituting $v_{j}(0)$ into (8) yields

(14) $$t_{jk}(m_{j})=\frac{1}{C_{jk}}\ln\left(\frac{D_{jk}}{E_{jk}}\right),$$

where

$$\begin{array}{rcl} C_{jk} & = & g_{ad}m_{j}+g_{L}+g_{E}d_{j},\\ D_{jk} & = & g_{ad}m_{j}(V_{jk}-V_{K})+g_{L}(V_{jk}-V_{L})+d_{j}g_{E}V_{jk} \end{array}$$

and

$$E_{jk}=g_{ad}m_{j}(\theta_{I}-V_{K})+g_{L}(\theta_{I}-V_{L})+d_{j}g_{E}\theta_{I}.$$

To compute $t_{1k}(h)$, we plug $V_{1k}$ into (9) and solve for $v_{1}(\tau )=\theta_{I}$. Recall that $m_{p_{\infty }}(v)=0$ if $v<V_{mp}$ and $m_{p_{\infty }}(v)=1$ if $v>V_{mp}$, as reflected in the piecewise formulation of (9). Thus, this calculation yields two terms, one corresponding to the time before *v* reaches $V_{mp}$ and one to the time after, namely

(15) $$t_{1k}(h)=\frac{1}{C_{1ka}}\ln\left(\frac{D_{1ka}}{E_{1ka}}\right)+\frac{1}{C_{1kb}}\ln\left(\frac{D_{1kb}}{E_{1kb}}\right),$$

where

$$\begin{array}{rcl} C_{1ka} & = & g_{L}+g_{E}d_{1},\\ D_{1ka} & = & g_{L}(V_{1k}-V_{L})+g_{E}d_{1}V_{1k},\\ E_{1ka} & = & g_{L}(V_{mp}-V_{L})+g_{E}d_{1}V_{mp},\\ C_{1kb} & = & g_{Nap}h+g_{L}+g_{E}d_{1},\\ D_{1kb} & = & g_{Nap}h(V_{mp}-V_{Na})+g_{L}(V_{mp}-V_{L})+g_{E}d_{1}V_{mp},\\ E_{1kb} & = & g_{Nap}h(\theta_{I}-V_{Na})+g_{L}(\theta_{I}-V_{L})+g_{E}d_{1}\theta_{I}. \end{array}$$

Now, cell 1 will either win or lose the race, if either $t_{1k}(h)<t_{jk}(m_{j})$ or $t_{1k}(h)>t_{jk}(m_{j})$, respectively. Each equation $t_{1k}(h)=t_{jk}(m_{j})$ defines a curve in the $\left(h,m_{j}\right)$ plane, which we denote as $C_{1j}$. These curves are also shown in Figure 3, where we numerically solved for $C_{12}$ and $C_{13}$. Note that points above the curve $C_{1j}$ correspond to cell 1 winning the race and points below this curve correspond to cell *j* winning the race.

### 3.4 Predicting jumping sequences

We now construct six 2D maps, $\Pi_{ij}$, that allow us to predict the order in which the cells jump up and down, to and from the active phase. To explain what these maps are, suppose that *i*,*j* and *k* are the cells’ distinct indices and, for convenience, temporarily let $s_{1}=h$, $s_{2}=m_{2}$, $s_{3}=m_{3}$ denote the slow variables for the three cells. If, at some time, cell *i* jumps down and cell *k* jumps up, then we will define a map $\Pi_{ik}$ from the $\left(s_{j},s_{k}\right)$ phase plane to the $\left(s_{i},s_{j}\right)$ phase plane that gives the position of $\left(s_{i},s_{j}\right)$ when cell *k* jumps down. We can determine the next cell to jump up, once cell *k* jumps down, by comparing the position of $\Pi_{ik}(s_{j},s_{k})$ to that of $C_{ij}$. For example, suppose that cell 1 jumps down. Then either cell 2 or cell 3 will jump up depending on whether $\left(m_{2},m_{3}\right)$ lies above or below the curve $C_{23}$, respectively. If cell 2 jumps up, then the map $\Pi_{12}(m_{2},m_{3})$ gives the position of $\left(h,m_{3}\right)$ when cell 2 jumps down. This position, in turn, determines whether cell 1 or cell 3 is the next cell to jump up; that is, cell 1 or cell 3 is the next cell to jump up if $\left(h,m_{3})=\Pi_{12}(m_{2},m_{3}\right)$ lies above or below $C_{13}$, respectively. Continuing in this way - comparing the output of the maps to the location of curves $C_{ij}$ - we can determine the cells’ jumping sequences.

We derive explicit formulas for the six maps $\Pi_{ij}$. The first step is to determine the value of the slow variable for cell *i* when cell *i* jumps down. We claim that there exist unique constants $s_{i}^{*}$ so that cell *i* jumps down when $s_{i}=s_{i}^{*}$; see Figure 2, where $s_{1}^{*}=h^{*}$, $s_{2}^{*}=m_{2}^{*}$ and $s_{3}^{*}=m_{3}^{*}$. These constants exist and are unique because: (i) cell *i* jumps down when it is in the active phase with $v_{i}=\theta_{I}$; (ii) while cell *i* is in the active phase, $\left(v_{i},s_{i}\right)$ lies along the right branch of the $v_{i}$-nullcline, $\left\{(v_{i},s_{i}):F_{i}(v_{i},s_{i})-g_{E}d_{i}(v_{i}-V_{E})=0\right\}$; and (iii) each of these right branches is monotone increasing or decreasing. This last statement can be verified for the concrete model (1) given in Section 2 by explicitly solving for each $s_{i}$ in terms of $v_{i}$. However, this monotonicity is also present in most reduced models for neuronal activity.

We now resume using *h*, $m_{2}$, $m_{3}$ to denote the slow variables for the three cells. First suppose that cell 1 is active; it will jump down when $h=h^{*}$. Let us say that this occurs at time τ=0, with $m_{j}=m_{j}(0)$ for j=2,3, and that cell *k*, k=2 or 3, wins the race and jumps up next; note that since *τ* is the slow time, τ=0 continues to hold throughout the jump. While cell *k* is up, *h* will increase, $m_{k}$ will increase, and $m_{j}$, j≠k, will decrease, governed by Equation (3). This state will persist until $m_{k}$ reaches $m_{k}^{*}$. From the active component of Equation (5) or (6), we can solve $m_{k}(\tau )=m_{k}^{*}$ to compute the slow time $T_{k}^{A}$ for which cell *k* remains active,

$$T_{k}^{A}=\frac{1}{\nu_{R}}\ln\left(\frac{1-m_{k}(0)}{1-m_{k}^{*}}\right),$$

where $\nu_{R}\in \{\lambda_{R},\mu_{R}\}$ as appropriate. While cell *k* is active, *h* is given by the silent part of Equation (4) with $h(0)=h^{*}$ and $m_{j}$, j≠k, is given by the silent part of Equation (5) or (6). From these equations, we can evaluate $h(T_{k}^{A})$, $m_{j}(T_{k}^{A})$, and we define the map $\Pi_{1k}$ by

$$\Pi_{1k}\left(m_{j}(0),m_{k}(0)\right)=\left(h\left(T_{k}^{A}\right),m_{j}\left(T_{k}^{A}\right)\right).$$

Specifically,

(16) $$\begin{array}{rcl} \Pi_{12}\left(m_{2}(0),m_{3}(0)\right) & = & \left(h\left(T_{2}^{A}\right),m_{3}\left(T_{2}^{A}\right)\right)\\ & = & \left(1+\left(h^{*}-1\right)\Gamma_{2}^{\sigma_{L}/\lambda_{R}},m_{3}(0)\Gamma_{2}^{\mu_{L}/\lambda_{R}}\right) \end{array}$$

and

(17) $$\begin{array}{rcl} \Pi_{13}\left(m_{2}(0),m_{3}(0)\right) & = & \left(h\left(T_{3}^{A}\right),m_{2}\left(T_{3}^{A}\right)\right)\\ & = & \left(1+\left(h^{*}-1\right)\Gamma_{3}^{\sigma_{L}/\mu_{R}},m_{2}(0)\Gamma_{3}^{\lambda_{L}/\mu_{R}}\right), \end{array}$$

where

(18) $$\Gamma_{j}=\left(1-m_{j}^{*}\right)/\left(1-m_{j}(0)\right)$$

for j∈{2,3}.

If the output of $\Pi_{1k}$ in the $\left(h,m_{j}\right)$ plane is above or below the curve $C_{1j}$, then cell 1 or cell *j* jumps up after cell *k*, respectively. Similarly, if we apply $\Pi_{1k}$ to the entire region in the positive (j,k) quadrant lying below curve $C_{jk}$, corresponding to cell *k* jumping after cell 1, then we can determine which, if any, initial $\left(m_{j},m_{k}\right)$ cause cell *j* to jump after cell *k* and which, if any, lead to cell 1 jumping after cell *k*. Note that for analyzing possible repetitive solutions, we really only need to consider inputs to $\Pi_{1k}$ that satisfy

(19) $$0\leq m_{2}\leq m_{2}^{*},\;0\leq m_{3}\leq m_{3}^{*}.$$

This constraint is appropriate because if, for example, $m_{2}>m_{2}^{*}$, then once cell 2 is released from inhibition and jumps up, it can never reach the threshold $v_{2}=\theta_{I}$.

Using a similar approach, based on computing an active time from the active component of one of the Equations (4), (5), and (6) and tracking the evolution of the slow variables of the two silent cells with the silent parts of the remaining two equations from this set, the maps $\Pi_{ij}$ can be defined for each combination of i≠j from {1,2,3}. The map $\Pi_{ij}$ takes values of the slow variables of cells *j* and *k*, i≠j≠k, as inputs, and gives values of the slow variables of cells *i* and *k* as outputs. In particular, for each pair j,k∈{2,3} with j≠k, we have

(20) $$\begin{array}{rcl} \Pi_{jk}\left(h(0),m_{k}(0)\right) & = & \left(h\left(T_{k}^{A}\right),m_{j}\left(T_{k}^{A}\right)\right)\\ & = & \left(1+\left(h(0)-1\right)\Gamma_{k}^{\sigma_{L}/\nu_{R}},m_{j}^{*}\Gamma_{k}^{\omega_{L}/\nu_{R}}\right) \end{array}$$

and

(21) $$\begin{array}{rcl} \Pi_{j1}\left(h(0),m_{k}(0)\right) & = & \left(m_{j}\left(T_{1}^{A}\right),m_{k}\left(T_{1}^{A}\right)\right)\\ & = & \left(m_{j}^{*}{\left[\frac{h^{*}}{h(0)}\right]}^{\omega_{L}/\sigma_{R}},m_{k}(0){\left[\frac{h^{*}}{h(0)}\right]}^{\nu_{L}/\sigma_{R}}\right), \end{array}$$

where $\Gamma_{k}$ is defined in (18) and where (ν,ω)=(λ,μ) if k=2 while (ν,ω)=(μ,λ) if k=3. As previously, we can bound the ranges of the slow variables that are relevant for repeated states, using (19) and

(22) $$h^{*}\leq h\leq 1.$$

If cell *i* jumps down at time 0 and the inputs to the map specify that cell *j* jumps next, then the location of the coordinate determined by the outputs of $\Pi_{ij}$, relative to the curve $C_{ik}$, determines whether cell *i* or cell *k* will follow cell *j* into the active phase.

Taken collectively, the curves and maps defined in this section gives us a complete view of the possible jump sequences that system (1) can generate, at least if *ϵ* is small enough to justify the fast-slow decomposition that we have used. Consider the regions in the $\left(m_{2},m_{3}\right)$, $\left(h,m_{2}\right)$, and $\left(h,m_{3}\right)$ phase planes that satisfy (19) and (22). Within the $\left(m_{2},m_{3}\right)$ plane, assume that the curve $C_{23}$ intersects the relevant region; otherwise, cell 1 will always be followed by the same other cell. The map $\Pi_{12}$ takes the region above the curve to a set in the $\left(h,m_{3}\right)$ plane and the map $\Pi_{13}$ takes the region below the curve to a set in the $\left(h,m_{2}\right)$ plane, with similar actions for $\Pi_{21}$, $\Pi_{23}$, $\Pi_{31}$, $\Pi_{32}$ on the other planes. Since the solutions to the ODEs we consider are continuous in initial conditions, the maps take connected regions into connected regions, and thus we only need to consider the actions of the maps on the regions’ boundaries in order to determine the possible next outcomes from a given starting point. For a particular parameter set, repeated iteration of the maps may show convergence to a single attracting jump sequence or may otherwise constrain the jump orders that are possible. Alternatively, inverses of the maps can be easily defined using the backwards flow of the ODEs, and repeated iterations of the inverses of the maps, applied to some selected region in one of the phase planes, show which sets contain initial conditions that could end up in the selected region.

### 3.5 Numerical examples

We now use numerical computations, performed with MATLAB and XPPAUT (http://www.pitt.edu/~phase), to illustrate the theory from the previous subsections. Figure 3 shows curves and regions in each of the 2D phase planes associated with pairs of slow variables of model (1). These structures were generated by starting from the full model, with function and parameter values given in the Appendix (see Table 1), and making the simplifying assumptions described above for the ϵ=0 limit (including adjusting $\theta_{m}$ to −54 mV from −50 mV to compensate for the switch from a smooth function to a Heaviside in the singular limit). In each panel, the relevant region can be defined using (19), (22), and the dashed straight line segments are boundaries of this region, each corresponding to $h^{*}$, $m_{2}^{*}$, or $m_{3}^{*}$. Within each region, there is a curve $C_{ij}$ that separates initial conditions that lead to different jumping outcomes, as discussed above. These curves are drawn in the same color as the boundary lines. For example, in Figure 3A, the solid blue curve in the $\left(m_{2},m_{3}\right)$ plane is $C_{23}$. If $\left(m_{2},m_{3}\right)$ lies in the region $R_{12}$, bounded below by $C_{23}$, above by the dashed blue line, and to the left by the $m_{3}$-axis, at the moment when cell 1 jumps down, then cell 2 jumps up next and $\Pi_{12}(m_{2},m_{3})$ is defined, while a value of $\left(m_{2},m_{3}\right)$ in the analogous region $R_{13}$ below $C_{23}$ yields a jump by cell 3, characterized by $\Pi_{13}(m_{2},m_{3})$. Similar regions are indicated in black in the $\left(h,m_{2}\right)$ plane in Figure 3B and in red in the $\left(h,m_{3}\right)$ plane in Figure 3C.

Consider again the $\left(m_{2},m_{3}\right)$ plane shown in Figure 3A. The region $R_{12}$ is mapped by $\Pi_{12}$ to a connected region in the $\left(h,m_{3}\right)$ plane. In Figure 3C, we represent part of the boundary of $\Pi_{12}(R_{12}):=\{\Pi_{12}(m_{2},m_{3}):(m_{2},m_{3})\in R_{12}\}$ with blue curves, carrying over the coloring of $R_{12}$ from Figure 3A. Similarly, a region $R_{32}$ below $C_{12}$ in the $\left(h,m_{2}\right)$ plane in Figure 3B also yields jumping by cell 2 and is mapped by $\Pi_{32}$ to a connected region in the $\left(h,m_{3}\right)$ plane. We indicate this region with black boundary curves in Figure 3C, carrying over the coloring from Figure 3B. The regions outlined in black and blue in the $\left(h,m_{3}\right)$ plane share a common boundary, corresponding to the condition that $\left(h,m_{3})=(h^{*},m_{3}^{*}\right)$ when cell 2 jumps up. We use a dashed black line to denote this common boundary in Figure 3C (by arbitrary convention, we color the dashed line to match the upper set). Now, the entire regions outlined in blue and black in the $\left(h,m_{3}\right)$ plane lie below the red curve $C_{13}$ (Figure 3C). Thus, we immediately know that, no matter what happened before, cell 3 will win the race and jump up when cell 2 jumps down. Similarly, in the $\left(m_{2},m_{3}\right)$ plane shown in Figure 3A, the black-bounded region $\Pi_{31}(R_{31})$ and the red-bounded region $\Pi_{21}(R_{21})$ lie entirely below $C_{23}$, and therefore cell 3 will always jump up after cell 1 as well.

The interesting case in this example arises in the $\left(h,m_{2}\right)$ plane. There, $\Pi_{13}(R_{13})$, outlined in solid blue and dashed red, and $\Pi_{23}(R_{23})$, outlined in solid and dashed red, are both intersected by $C_{12}$. Hence, there are initial conditions in our relevant regions for which the jump sequence 1,3,1 occurs and others for which the jump sequence 1,3,2 occurs, and similarly, there are initial conditions leading to jump sequences 2,3,1 and 2,3,2 as well. We can now summarize all possible jump sequences for the parameter set used in this example:

$$1\begin{array}{c} \to \\ \leftarrow \end{array}3\begin{array}{c} \to \\ \leftarrow \end{array}2,$$

possibly discarding a brief transient.

We selected various values of $\left(m_{2},m_{3}\right)$ constrained by (19) and we used each as an initial condition, assuming that cell 1 jumped down from the active phase at time 0. From each starting point, we repeatedly solved for the times involved in the race to jump up, using Equations (11), (14), and (15). We found that the trajectory emerging from each initial condition converged to the same attractor, with a jump sequence 13231323… . This attractor is illustrated with filled circles in Figure 3; the black circle in Figure 3A is mapped by $\Pi_{13}$ to the blue circle in Figure 3B, which is mapped by $\Pi_{32}$ to the black circle in Figure 3C, which is mapped by $\Pi_{23}$ to the red circle in Figure 3B, which is mapped by $\Pi_{31}$ back to the original black circle in Figure 3A. Note that the next jump predicted by the location of each circle matches that which actually occurs. Also, a subtle point arises because the *h* coordinate of the red circle is large. From this starting point, when cell 1 jumps up, it spends a long time in the active phase (large $T_{1}^{A}$), almost as long as if it started from h=1. During this time, trajectories in the $\left(m_{2},m_{3}\right)$ plane with $m_{3}(0)=m_{3}^{*}$ and different initial values of $m_{2}$ get compressed; see Equation (5). Thus, the black circle in Figure 3A ends up very close to the corner of the black region, which corresponds to $\Pi_{31}(1,m_{2}^{*})$.

We also performed direct numerical simulations of system (1), using steep but smooth sigmoidal functions instead of Heaviside functions for $m_{\infty }(v)$, $n_{\infty }(v)$, and $S_{\infty }(v)$, as described in the Appendix. These simulations also gave a 13231323…firing pattern, as predicted by the analysis. We defined firing transitions in these simulations using voltage decreases through −33 mV (the half-activation of the synaptic function $S_{\infty }(v)$ was set to −32 mV to agree with $\theta_{I}$). We allowed the system to converge to its stable firing pattern and then plotted the slow variable coordinates at these firing transitions as open circles in the corresponding panels of Figure 3. These coordinates agree well with the singular limit analysis.

In addition to the solid and open circles corresponding to the attractors in the singular limit and full simulations, respectively, certain points associated with transients are also plotted in Figure 3. An example of a transient 1,3,1,3 firing sequence found with the singular limit formulas, which led to a subsequent 2313231323…activation pattern, is marked with the blue asterisks in Figure 3A,B. In this example, initial conditions were chosen such that cell 1 jumped down with $\left(m_{2},m_{3})=(0.29,0.6\right)$, indicated by the rightmost asterisk in Figure 3A (label 1). Since the asterisk is below the blue solid curve $C_{23}$ in the plane shown, cell 3 jumps next. Obviously, the image of the initial point under $\Pi_{13}$ must lie in the range of $\Pi_{13}$ in the $\left(h,m_{2}\right)$ plane, which is bounded to the left, below and to the right by solid blue curves and above by a dashed red curve. We observe (Figure 3B, label 2) that this image lies at about $\left(h,m_{2})=(0.51,0.24\right)$, which is indeed in the relevant region but also is above the black solid curve $C_{12}$, meaning that cell 1 jumps up next. The image of $\left(h,m_{2}\right)$ under $\Pi_{31}$ is marked by the other asterisk in Figure 3A (label 3), which lies below $C_{23}$ such that cell 3 jumps again after cell 1. Finally, the image of that point under $\Pi_{13}$ is labeled by the other asterisk in Figure 3B (label 4); since that point is below the black curve $C_{12}$, cell 2 finally gets to fire after this second activation of cell 3.

We also obtained a similar 1,3,1,3 transient in full model simulations corresponding to the singular limit analysis. To match the singular limit, we used $\left(m_{2},m_{3})=(0.29,0.6\right)$ as our initial condition, with $v_{1}=-33\text{ mV}$ and $h=h^{*}$ such that time 0 represented the beginning of the jump down of cell 1. This point and the slow variable values at the next 3 jump down transitions are marked with red open squares in Figure 3. By construction, the red open square at label 1 lies in the same position as the blue asterisk there. The rest of these markers, near labels 2,3,4, lie quite close to the blue asterisks, showing that, in addition to correctly predicting the jumping sequence, the singular limit analysis gives good estimates to the slow variable values at jumping times in the original system, although the agreement is not perfect since *ϵ* is nonzero in the original system and our analysis replaces sigmoidal activation and coupling functions by step functions.

## 4 From six maps to one

### 4.1 Derivation of the map

We now present a somewhat different approach. Previously, we considered the six separate maps between the three different 2D slow phase planes, $\left(h,m_{2}\right)$, $\left(h,m_{3}\right)$, and $\left(m_{2},m_{3}\right)$. Here, we demonstrate that it is possible to use these six maps to reduce the dynamics to a single map, defined from some subset of the $\left(m_{2},m_{3}\right)$ phase plane into itself. Moreover, with some simplifying assumptions, we will derive an explicit formula for the map.

First, fix $\left(m_{2},m_{3}\right)$ and assume that when τ=0, cells 2 and 3 lie in the silent phase with $m_{2}(0)=m_{2}$ and $m_{3}(0)=m_{3}$. Suppose also that cell 1 lies in the active phase with $v_{1}(0)=\theta_{I}$, so that cell 1 jumps down at this time. Then either cell 2 or cell 3 will jump up. These two cells may take turns firing, but we assume that eventually, cell 1 will win a race and successfully jump up to the active phase again, from which it will subsequently jump down and start a new cycle. Choose T>0 to be the first time (after τ=0) that cell 1 jumps down. Then define a map as simply

(23) $$\Pi (m_{2},m_{3})=\left(m_{2}(T),m_{3}(T)\right).$$

In other words, iterates of Π keep track of the positions of $\left(m_{2},m_{3}\right)$ every time that cell 1 jumps down from the active phase.

We can obtain explicit formulas for this map if we assume that the slow variables satisfy (4), (5), and (6). Different sets of formulas will be relevant on the regions $R_{12}$ or $R_{13}$, above or below $C_{23}$ respectively, corresponding to whether cell 2 or cell 3 wins the race and jumps up first when cell 1 jumps down. We can subdivide each of these regions based on the number of times that cells 2 and 3 take turns firing after cell 1 jumps down, before cell 1 jumps up again. On each of these subregions of the $\left(m_{2},m_{3}\right)$ phase plane, a different formula applies. Here we derive the formulas for the case in which cell 2 jumps up at τ=0 when cell 1 jumps down. Formulas for the case in which cell 3 jumps up at τ=0 are derived in a similar manner. First we derive the formulas for the map Π and then determine for which region of the $\left(m_{2},m_{3}\right)$ phase plane each component of the formula is valid.

Recall that cells 2 and 3 may take turns firing for 0≤τ<T. Let $N_{2}$ and $N_{3}$ be the number of times that cells 2 and 3, respectively, jump up during this time interval. We note that either the two cells fire the same number of times, in which case $N_{3}=N_{2}$, or cell 2 fires one more time than cell 3, in which case $N_{3}=N_{2}-1$. Using the definitions and notation described in the preceding section, we find that:

If $N_{3}=N_{2}$, then

$$\Pi (m_{2},m_{3})=\Pi_{31}\circ \Pi_{23}\circ {\left(\Pi_{32}\circ \Pi_{23}\right)}^{N_{2}-1}\circ \Pi_{12}(m_{2},m_{3}).$$

If $N_{3}=N_{2}-1$, then

$$\Pi (m_{2},m_{3})=\Pi_{21}\circ {\left(\Pi_{32}\circ \Pi_{23}\right)}^{N_{2}-1}\circ \Pi_{12}(m_{2},m_{3}).$$

We derive explicit formulas for these maps using the formulas for $\Pi_{ij}$ derived in the preceding section. In what follows, we use the notation $\Pi (m_{2},m_{3})=({\hat{m}}_{2},{\hat{m}}_{3})$, and we employ the time constants $\sigma_{L}$, $\sigma_{R}$, $\lambda_{L}$, $\lambda_{R}$, $\mu_{L}$ and $\mu_{R}$ introduced in Section 3.2. The formulas are derived by direct calculations; we first consider two simple cases, before presenting the general formulas. For these formulas, recall that $h^{*}$ denotes the value of *h* attained when cell 1 is about to jump down (i.e., cell 1 is active, cell 1 is not inhibited, and $v_{1}=\theta_{I}$, see Figure 2A); similarly, $m_{2}^{*}$, $m_{3}^{*}$ denote the values of $m_{2}$, $m_{3}$ when cell 2 or cell 3 is about to jump down ($v_{2}=\theta_{I}$, $v_{3}=\theta_{I}$), respectively.

*Case 1:*$N_{2}=1$*,*$N_{3}=0$*.*

Here $\left({\hat{m}}_{2},{\hat{m}}_{3})=\Pi_{21}\circ \Pi_{12}(m_{2},m_{3})=\Pi_{21}(h^{1},m_{3}^{1}\right)$, where

(24) $$\begin{array}{rl} h^{1} & =1+\left(h^{*}-1\right){\left(\frac{1-m_{2}^{*}}{1-m_{2}}\right)}^{\sigma_{L}/\lambda_{R}},\;m_{3}^{1}=m_{3}{\left(\frac{1-m_{2}^{*}}{1-m_{2}}\right)}^{\mu_{L}/\lambda_{R}},\\ {\hat{m}}_{2} & =m_{2}^{*}{\left(\frac{h^{*}}{h^{1}}\right)}^{\lambda_{L}/\mu_{R}},\;{\hat{m}}_{3}=m_{3}^{1}{\left(\frac{h^{*}}{h^{1}}\right)}^{\mu_{L}/\lambda_{R}}. \end{array}$$

To achieve $N_{3}=0$, we need that cell 1, not cell 3, jumps up when cell 2 jumps down. From the earlier discussion, this is true if $\left(h^{1},m_{3}^{1}\right)$ lies above the curve $C_{13}$. Together with (24), this criterion leads to a condition on $\left(m_{2},m_{3}\right)$, which defines a region in the $\left(m_{2},m_{3}\right)$ plane where this case occurs. One could numerically compute this region using the definition of $C_{13}$ given in the preceding section. Alternatively, we will now make a simplifying assumption that allows us to compute this region analytically. The validity of this assumption will be confirmed by comparing the firing sequence of the full model with that predicted by the analysis in the examples in the following section.

Our simplifying assumption can be described as follows: Suppose that at some time, say t=0, cell 1 lies in the silent phase and is released from inhibition (by either cell 2 or cell 3). We assume that the time it takes cell 1 to jump up and reach the threshold $\theta_{I}$ is independent of h(0). It follows from this assumption that the curves $C_{12}$ and $C_{13}$ are horizontal; that is, they can be written as $m_{2}=M_{2}$ and $m_{3}=M_{3}$ for some constants $M_{2}$ and $M_{3}$.

Using this assumption, we conclude that Case 1 occurs if: (a) $\left(m_{2},m_{3}\right)$ lies above $C_{23}$ (so that cell 2 jumps up when cell 1 jumps down), and (b) $m_{3}^{1}>M_{3}$, which, together with (24), gives

(25) $$m_{3}>M_{3}{\left(\frac{1-m_{2}}{1-m_{2}^{*}}\right)}^{\mu_{L}/\lambda_{R}}\equiv k_{1}^{2}(m_{2}).$$

We define the curve $K_{1}^{2}$ by

$$K_{1}^{2}:=\left\{(m_{2},m_{3})\in R_{12}:m_{3}=k_{1}^{2}(m_{2})\right\}.$$

Here, the superscript ‘2’ reflects that cell 2 jumps up when cell 1 jumps down, while the subscript ‘1’ corresponds to the number of jumps that follow before cell 1 jumps up again (i.e., $N_{2}+N_{3}=1$). There is another curve, given by $m_{2}=K_{1}^{3}(m_{3})$, corresponding to cell 3 jumping up when cell 1 jumps down. The formula for $K_{1}^{3}$ is derived in a similar manner, and $K_{1}^{3}\subset R_{13}$, below $C_{23}$.

*Case 2:*$N_{2}=1$*,*$N_{3}=1$*.*

**Fig. 4:**
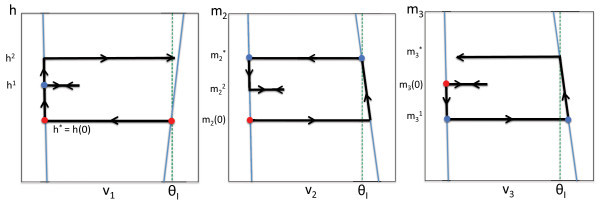
Phase planes for Case 2. We start at the red disc, when cell 1 jumps down from the active phase (or equivalently, with respect to the slow time *τ*, when cell 1 enters the silent phase). At this time, cell 2 wins the race and jumps up. When cell 2 jumps down, cell 3 wins the race with cell 1 and jumps up. Finally, when cell 3 jumps up, cell 1 wins the race with cell 2 and jumps up.

This case is illustrated in Figure 4. Here,

$$({\hat{m}}_{2},{\hat{m}}_{3})=\Pi_{31}\circ \Pi_{23}\circ \Pi_{12}(m_{2},m_{3})=\Pi_{31}\circ \Pi_{23}\left(h^{1},m_{3}^{1}\right)=\Pi_{31}\left(h^{2},m_{2}^{2}\right),$$

where

(26) $$\begin{array}{rl} h^{2} & =1+\left(h^{1}-1\right){\left(\frac{1-m_{3}^{*}}{1-m_{3}^{1}}\right)}^{\sigma_{L}/\mu_{R}},\;m_{2}^{2}=m_{2}^{*}{\left(\frac{1-m_{3}^{*}}{1-m_{3}^{1}}\right)}^{\lambda_{L}/\mu_{R}},\\ {\hat{m}}_{2} & =m_{2}^{2}{\left(\frac{h^{*}}{h^{2}}\right)}^{\lambda_{L}/\sigma_{R}},\;{\hat{m}}_{3}=m_{3}^{*}{\left(\frac{h^{*}}{h^{2}}\right)}^{\mu_{L}/\sigma_{R}}, \end{array}$$

where $h^{1}$, $m_{3}^{1}$ are defined in (24). For this case to occur, we need that: (i) cell 3 jumps up when cell 2 jumps down, and (ii) cell 1 jumps up when cell 3 jumps down. These conditions are satisfied if: (i) $\left(h^{1},m_{3}^{1}\right)$ lies below the curve $C_{13}$, and (ii) $\left(h^{2},m_{2}^{2}\right)$ lies above the curve $C_{12}$. These conditions define a region in the $\left(m_{2},m_{3}\right)$ phase plane. If we make the same assumption as in Case 1, that the curves $C_{12}$ and $C_{13}$ are given by $m_{2}=M_{2}$ and $m_{3}=M_{3}$ for some constants $M_{2}$ and $M_{3}$, then Case 2 occurs if: (a) $\left(m_{2},m_{3}\right)$ lies above $C_{23}$ (i.e., in $R_{12}$), (b) $m_{3}^{1}<M_{3}$, and (c) $m_{2}^{2}>M_{2}$. It follows from (24) and (26) that (b) and (c) are satisfied if $k_{2}^{2}(m_{2})<m_{3}<k_{1}^{2}(m_{2})$ where

(27) $$k_{2}^{2}(m_{2}):=\left[\frac{1-{\left(\frac{m_{2}^{*}}{M_{2}}\right)}^{\mu_{R}/\lambda_{L}}(1-m_{3}^{*})}{{\left(1-m_{2}^{*}\right)}^{\mu_{L}/\lambda_{R}}}\right]{\left(1-m_{2}\right)}^{\mu_{L}/\lambda_{R}}.$$

Furthermore, we define the boundary curve

$$K_{2}^{2}=\left\{(m_{2},m_{3})\in R_{12}:m_{3}=k_{2}^{2}(m_{2})\right\},$$

such that Case 2 corresponds to those $(m_{2},m_{3})\in R_{12}$ between $K_{1}^{2}$ and $K_{2}^{2}$. 

*General case*: The general formulas are derived recursively, again by direct calculation. Let

(28) $$\begin{array}{rcl} \alpha & = & m_{2}^{*}{\left(1-m_{3}^{*}\right)}^{\lambda_{L}/\mu_{R}},\;\beta =m_{3}^{*}{\left(1-m_{2}^{*}\right)}^{\mu_{L}/\lambda_{R}},\\ f(m) & = & \alpha {\left(1-m\right)}^{-\lambda_{L}/\mu_{R}},\;g(m)=\beta {\left(1-m\right)}^{-\mu_{L}/\lambda_{R}},\\ m_{2}^{k} & = & {\left(f\circ g\right)}^{k}(m_{2})\;\text{if }k\text{ is even},\\ m_{3}^{k} & = & {\left(g\circ f\right)}^{k-1}\left(m_{3}^{1}\right)\;\text{if }k\text{ is odd},\\ a_{k} & = & \left\{ \begin{array}{ll} {\left(\frac{1-m_{2}^{*}}{1-m_{2}^{k-2}}\right)}^{\sigma_{L}/\lambda_{R}} & \text{if }k\text{ is odd }\left(\text{here, we let }m_{2}^{-1}=m_{2}\right),\\ {\left(\frac{1-m_{3}^{*}}{1-m_{3}^{k-2}}\right)}^{\sigma_{L}/\mu_{R}} & \text{if }k\text{ is even}, \end{array}\right.\\ h^{0} & = & 1+\left(h^{*}-1\right)a_{1}, \end{array}$$

(29) $$h^{k}=a_{k+1}h^{k-1}+1-a_{k+1}\;\text{for }k\geq 1,$$

and $N=N_{2}+N_{3}$. Then $\Pi (m_{2},m_{3})=({\hat{m}}_{2},{\hat{m}}_{3})$, where

(30) $${\hat{m}}_{2}=\left\{ \begin{array}{ll} m_{2}^{*}{\left(\frac{h^{*}}{h^{N}}\right)}^{\lambda_{L}/\sigma_{R}} & \text{if }N_{2}=N_{3},\\ m_{2}^{N}{\left(\frac{h^{*}}{h^{N}}\right)}^{\lambda_{L}/\sigma_{R}} & \text{if }N_{2}\neq N_{3}, \end{array}\right.$$

and

(31) $${\hat{m}}_{3}=\left\{ \begin{array}{ll} m_{3}^{N}{\left(\frac{h^{*}}{h^{N}}\right)}^{\mu_{L}/\sigma_{R}} & \text{if }N_{2}=N_{3},\\ m_{3}^{*}{\left(\frac{h^{*}}{h^{N}}\right)}^{\mu_{L}/\sigma_{R}} & \text{if }N_{2}\neq N_{3}. \end{array}\right.$$

Formulas (30) and (31) hold only if cells 2 and 3 take turns firing $N_{2}$ and $N_{3}$ times, respectively, before cell 1 finally jumps up. As before, we can use the explicit formulas for $h^{k}$, $m_{2}^{k}$, $m_{3}^{k}$ to derive explicit conditions on the initial point $\left(m_{2},m_{3}\right)$ for when this is true. We do not give the explicit general formula here. In the following section, we consider concrete examples and will give the formulas needed for the analysis of those examples.

### 4.2 Numerical examples

**Fig. 5:**
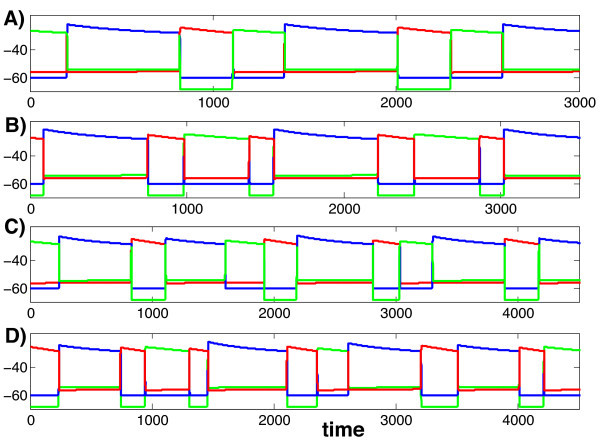
Four solutions of (1) for different values of the parameters $\left(\lambda_{L},\mu_{L}\right)$, given in the text. In each panel, the *blue*, *green* and *red curves* correspond to cells 1, 2, and 3, respectively.

Again, we use MATLAB and XPPAUT to illustrate our results numerically. Figure 5 shows four solutions of system (1), each generating a different firing pattern, corresponding to parameter values given in the Appendix in Table 2. The parameters for each of these solutions are exactly the same except for the rates $\lambda_{L}$ and $\mu_{L}$ at which the slow variables $m_{2}$ and $m_{3}$ decay while cells 2 and 3 lie in the silent phase. Here we show stable attractors so the firing patterns presented repeat as time evolves. In each panel, cells 1, 2, and 3 are displayed with the colors blue, green and red, respectively. We can denote the firing patterns shown in Figure 5A-D as (132), (1323), (13123132), and (132313213), respectively, in reference to the shortest firing pattern that repeats in each case. The analysis presented in Section 4.1 is very useful in understanding the origins of these firing patterns and how transitions between the firing patterns take place as parameters are varied. 

**Fig. 6:**
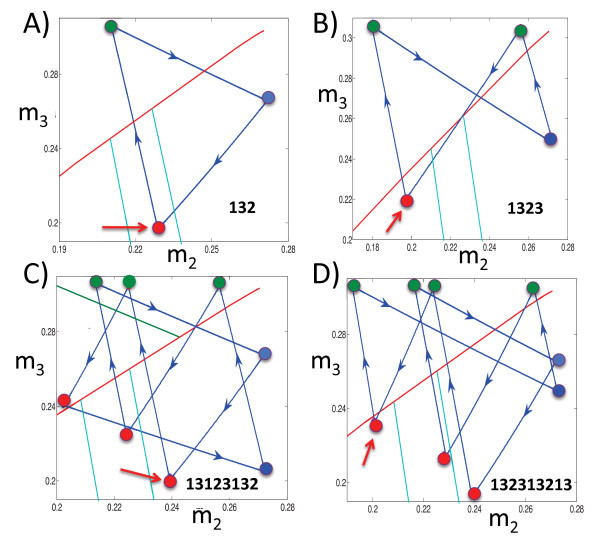
The projections of the solutions shown in Figure 5 onto the $\left(m_{2},m_{3}\right)$ phase plane. The *red curves* are $C_{23}$, while the *turquoise curves* are $K_{1}^{2}$ (larger $m_{2}$) and $K_{2}^{2}$ (smaller $m_{2}$). The *red*, *blue*, and *greencircles* correspond when cells 1, 2, and 3 jump down, respectively. The *red arrows* denote the starting points for the discussions of the panels in the text. Finally, the *numerical legend* within each panel indicates the firing sequence that repeats periodically.

Figure 6 shows the projections of the solutions exhibited in Figure 5 onto the $\left(m_{2},m_{3}\right)$ phase plane. First consider Figure 6A. The blue curve is the projection of the solution shown in Figure 5A onto the $\left(m_{2},m_{3}\right)$ phase plane. For this solution, $\left(\lambda_{L},\mu_{L})=(1/3\text{,}500,1/2\text{,}000\right)$. The red, blue, and green circles correspond to when cells 1, 2, and 3 jump down, respectively. The red curve corresponds to $C_{23}$ and the two turquoise curves correspond to $K_{1}^{3}$ (to the right of/above the red circle) and $K_{2}^{3}$ (to the left of/below the red circle), respectively. If we start at the red circle (at the arrow) and follow the blue trajectory, then we find that cells 1, 3, and 2 take turns firing, in that order. Note that when cell 1 jumps down, $\left(m_{2},m_{3}\right)$ lies below $C_{23}$, such that cell 3 jumps after cell 1, and $k_{2}^{3}(m_{3})<m_{2}<k_{1}^{3}(m_{3})$. This position corresponds to Case 2 above. As predicted by the theory for that case, when cell 1 jumps down, cell 3 jumps up and then cell 2 jumps up before cell 1 jumps up again. 

Next consider Figure 6B. Now $\left(\lambda_{L},\mu_{L})=(1/4\text{,}200,1/1\text{,}700\right)$. As before, when cell 1 jumps down at the red circle marked by the arrow, $\left(m_{2},m_{3}\right)$ lies below $C_{23}$, so cell 3 jumps up when cell 1 jumps down. However, now $m_{2}<k_{2}^{3}(m_{3})$. According to the theory, this relation implies that after cell 3 jumps down, cell 2 jumps up and down, and then cell 3 does the same again before cell 1 jumps up, as observed in the simulation. We note that for this example, $K_{3}^{3}(m_{3})<0$, so cell 3 can fire no more than two times between firings of cell 1. Note that the firing order of the attractor in Figures 5B and 6B, namely 1323, matches that shown in Figure 3.

For Figure 6C, $\left(\lambda_{L},\mu_{L})=(1/4\text{,}500,1/2\text{,}000\right)$. Once again, we start at the red circle indicated by the arrow when cell 1 jumps down. At that time, $\left(m_{2},m_{3}\right)$ lies below $C_{23}$ and above $K_{1}^{3}$; that is, $m_{2}>k_{1}^{3}(m_{3})$. Thus, we expect that cell 3 jumps up and then cell 1 jumps down again without any jumps by cell 2, and that is what is observed numerically along the trajectory from the initial red circle to the green circle to the next red circle (the 131 part of the solution). Now, this next red circle lies above $C_{23}$. Thus, the next cell to jump should be cell 2, as is seen in the figure by following the trajectory forward again. It turns out that at that second red circle, $k_{2}^{2}(m_{2})<m_{3}<k_{1}^{2}(m_{2})$ (not shown in the figure), which implies that cell 3 follows cell 2 before cell 1 jumps down yet again (the 231 part of the solution following the initial 131 part). Finally, when cell 1 jumps down for the third time, the corresponding red circle lies between $K_{2}^{2}$ and $K_{1}^{2}$, with $k_{2}^{2}(m_{3})<m_{2}<k_{1}^{2}(m_{3})$, as can be seen in Figure 6C. This relation implies that cell 3 and then cell 2 jump after cell 1, yielding the final 23 part of the solution before the trajectory returns to the initial red circle and the whole pattern repeats.

Finally, consider Figure 6D. Here, $\left(\lambda_{L},\mu_{L})=(1/4\text{,}500,1/1\text{,}800\right)$. As with each of these examples, the curves $C_{23}$, $K_{1}^{2}$ and $K_{2}^{2}$ (and similarly $K_{1}^{3}$, $K_{2}^{3}$ on the other side of $C_{23}$) divide the phase plane into separate regions. These regions determine how many times cells 2 and 3 take turns firing between the firings of cell 1.

## 5 Discussion

 We have presented a method for predicting the order with which model neurons or populations of synchronized neurons, arranged in a mutually inhibitory ring, will activate. We have derived and illustrated the method for a network of three cells, each with 2D intrinsic dynamics, motivated by models for rhythm-generating circuits in the mammalian respiratory brain stem [4-6]. Our approach involves the derivation of explicit formulas that can be used to partition reduced phase spaces into regions leading to different firing sequences. These ideas require a decomposition of dynamics into two distinct time scales. We have assumed an explicit fast-slow decomposition of the model equations for each neuron, into a fast voltage equation and a slow gating variable equation, with similar time scales present across all neurons, but we expect that the results would extend to other cases involving drift along slow manifolds alternating with fast jumps between manifolds yet lacking this explicit decomposition. A powerful aspect of the approach is that mapping from one activation to the next only requires evaluation of our formulas on a small number of curves in a particular reduced phase space. Moreover, if the images of these curves do not intersect the partition curves in the appropriate image space, then we can conclude that certain neurons will always become active in a fixed order, possibly after a short transient. Our formulas involve the time that it takes each neuron’s voltage to jump up to threshold upon release from inhibition. With the additional assumption that, for a particular cell in the network, this time does not depend on the cell’s slow variable in the silent phase, we obtain an especially strong result. That is, from a starting configuration with the distinguished cell at the end of an active phase, we arrive at a collection of closed form expressions that can be computed iteratively to determine, for all possible initial values of the other two cells’ slow variables, exactly how many times the other two cells will take turns activating before the distinguished cell activates again. We note that our additional assumption is reasonable for slow variables modulating currents that act predominantly to sustain or terminate activity. Finally, by observing the effects of parameters on the formulas that we obtain, we can determine how changes in parameters will alter model solutions, as we have demonstrated. 

 Interestingly, in the examples that we show and others that we have explored, the trajectories of the model system that we have considered tend to settle to one particular attractor for each parameter set. This lack of bistability likely stems from the fact that when each neuron is active, the other two neurons in the system experience a strong, common inhibitory signal, albeit with different strengths, and the fact that the neurons’ intrinsic dynamics is low-dimensional. It is well known that common inhibition can be strongly synchronizing in neuronal models (e.g., [1,2,14-18]). The model that we consider has rapid onset of inhibition, which prevents synchronization, but the strong inhibition is nonetheless able to quickly compress trajectories associated with different initial conditions towards similar paths through phase space. Perhaps evolutionary pressures conspire to steer dynamics of respiratory rhythm-generators away from regimes supporting bistability, to maintain a stable respiratory rhythm that adjusts smoothly to changes in environmental or metabolic demands. Other recent work has also been directed towards reduced descriptions that yield complete information about possible attractors in networks that are similar to the one we consider but tend to support multistability [19-21]. For example, trajectories can be generated for Poincaré maps based on phase lags, also under the assumption that units activate via release from inhibition, with fixed points corresponding to periodic states [22]. While that approach can handle high-dimensional dynamics and gives a rather complete description of how phase relations between units evolve, it requires that all cells fire before any cell fires twice and it is computationally intensive relative to our method, with additional computation needed for networks with strong coupling or significant asymmetries. 

 Previous work has presented analytical methods based on a fast-slow decomposition for solutions of model neuronal networks featuring two interacting populations, each synchronized, with different forms of intrinsic dynamics or two or more synchronized clusters of neurons within one population (e.g., [1,2,23-25]). The methods in this article provide tools for dealing with multiple different forms of dynamics. They are particularly well suited for three-population networks with 2D intrinsic dynamics as presented in this article, and a set of general assumptions that are sufficient for the method to apply are presented in the Appendix. In more complicated settings, the subspaces of slow variables that we consider would become higher-dimensional, such that while the same theory would apply, its application would be more cumbersome. Another direction for future consideration is the analysis of solutions in which suppressed neurons may escape from the silent phase, rather than being released from inhibition. Such solutions are qualitatively different than what we consider in this article, because the race to escape would take place within the slow dynamics. Similar issues have been considered previously in the context of the break-down of synchronization and the development of clustered solutions within a single population [21,25-27], and with simple slow dynamics, analysis of the race to escape among heterogeneous populations would be straightforward. Some networks may feature solutions involving some transitions by escape and some by release [6], however, and combining both effects, especially with adaptation that allows slow adjustment of inhibitory strength within phases [28,29], would be more complicated and remains for future study. Additional study would also be required to weaken the other assumptions we have made in our analysis. In particular, it might be possible to improve the quantitative agreement between our formulas and the actual slow variable values at jumps, and the actual jumping order for some parameter sets near transitions between solution types, by no longer treating sigmoidal activation and coupling functions as step functions; however, it is not clear how to derive explicit formulas without these approximations. Finally, it would be interesting to try to generalize our approach to noisy systems. Presumably, this generalization would involve replacing our boundary curves with distributions of jumping probabilities defined over regions of each slow variable space, leading to probabilistically defined jumping orders and mappings between spaces. 

## Appendix 1: Model details

In system (1), the functions $F_{i}$ are given by (2). Equations (1) and (2) involve several additional functions. The functions $x_{\infty }(v)=1/\{1+\exp[(v-\theta_{x})/\sigma_{x}]\}$ for $x\in \{h,m,m_{p},n\}$, while

(32) $$\tau_{i}(v)=\tau_{a,i}+\tau_{b,i}/\left\{1+\exp\left[\left(v-\theta_{i}^{\tau }\right)/\sigma_{i}^{\tau }\right]\right\},\;i\in \{h,2,3\}.$$

Parameter values for Equations (1) and (2) and for these additional functions are listed in Tables 1 and 2. These values were chosen by starting from those in published studies [6,8] and making changes to achieve interesting dynamics; also, we rescaled the capacitance *C* to 1 pF and divided all conductances by its original value, 20, correspondingly. Note that the actual values are not important as long as they give a certain nullcline structure and fast-slow time scale separation, as these do (see the general assumptions in Appendix 2 below).

Note that given $\left(\tau_{a,i},\tau_{b,i}\right)$, i=h,2,3, one can compute the *σ*, *λ*, and *μ* values that appear in (4), (5), and (6). That is, taking into account that $\theta_{2}^{\tau }$ and $\theta_{3}^{\tau }$ in Table 1 are well above the voltages actually achieved in our simulations and that $\sigma_{h}^{\tau }<0$, we compute the singular limit parameter values in the table as

$$\begin{array}{rcl} \sigma_{L} & = & \epsilon /\tau_{a,h},\;\sigma_{R}=\epsilon /(\tau_{a,h}+\tau_{b,h}),\\ \lambda_{L} & = & \epsilon /(\tau_{a,2}+\tau_{b,2}),\;\lambda_{R}=\epsilon /(\tau_{a,2}+\tau_{b,2}),\\ \mu_{L} & = & \epsilon /(\tau_{a,3}+\tau_{b,3}),\;\mu_{R}=\epsilon /(\tau_{a,3}+\tau_{b,3}). \end{array}$$

The parameter values listed in Table 1 for $\tau_{a,h}$, $\tau_{b,h}$ were used during times when cell 3 was in the active phase and in the subsequent races, while $\tau_{a,h}=5.75$, $\tau_{b,h}=-0.75$ were applied during times when cell 4 was active and in the subsequent races; similarly, $\sigma_{L}$ was changed to 1/575 when cell 4 was active. These values of $\tau_{a,h}$, $\tau_{b,h}$ were obtained from preliminary simulations using a slightly different form of $\tau_{h}(v)$ that had been used in earlier studies [6,8,30], which gave qualitatively identical behavior. This original $\tau_{h}(v)$ took different values depending on whether cell 3 or cell 4 was active because $v_{1}$ belonged to different intervals in the two cases. The form of $\tau_{h}(v)$ that we adopted, as given in Equation (32), was chosen to unify the form of the equations across all three neurons and to simplify numerical exploration of parameter space. We note that a change in $\theta_{m_{p}}$ from −50 to −52 changed the attractor from 13231323…to 132313213…as in Figure 6A, although this parameter set did not give the full range of patterns seen in the other panels of Figure 6.

Similarly, with the values of $\theta_{i}^{\tau }$, $\sigma_{i}^{\tau }$, i=h,2,3 given in Table 2, the singular limit parameter values in Table 2 are obtained from

$$\begin{array}{rcl} \sigma_{L} & = & \epsilon /\tau_{a,h},\;\sigma_{R}=\epsilon /(\tau_{a,h}+\tau_{b,h}),\\ \lambda_{L} & = & \epsilon /(\tau_{a,2}+\tau_{b,2}),\;\lambda_{R}=\epsilon /\tau_{a,2},\\ \mu_{L} & = & \epsilon /(\tau_{a,3}+\tau_{b,3}),\;\mu_{R}=\epsilon /\tau_{a,3}. \end{array}$$

For all panels in Figures 5 and 6, we used the parameter set in Table 2, except that we adjusted $\left(\tau_{a,2},\tau_{b,2},\tau_{a,3},\tau_{b,3}\right)$ for panels B,C,D. Specifically, we set $\left(\tau_{a,2},\tau_{b,2},\tau_{a,3},\tau_{b,3}\right)$ to (35,7,20,−3) in Figures 5B and 6B, (35,10,20,0) in Figures 5C and 6C, and (35,10,20,−2) in Figures 5D and 6D.

## Appendix 2: General assumptions

System (1) has certain properties that make it suitable for the analysis that we perform. Given a network of three synaptically coupled elements, our analysis can proceed if the following assumptions on the network and its dynamics are satisfied. 

(A1) Each unit in the network consists of a system of two ordinary differential equations (ODE), one for the evolution of a fast variable with an O(1) vector field, call it $f_{j}$, and one for a slow variable with an O(ϵ) vector field, $s_{j}$, for j∈{1,2,3}, where *ϵ* is a small, positive parameter.

(A2) Each unit is coupled to both of the other units in the network. The coupling from unit *j* to unit *k* appears as a Heaviside step function $H(f_{j}-\theta_{I})$, or a sufficiently steep increasing sigmoidal curve with half-activation $\theta_{I}$, in the ODE for $f_{k}$.

(A3) The fast vector field of each unit is a decreasing function of the strengths of the inputs that unit receives. Thus, if $f_{j}$ decreases through $\theta_{I}$, such that the input from unit *j* to the other units turns off, then $df_{k}/dt$ increases for k≠j.

(A4) When both inputs to unit *j* are fixed, the nullcline of its fast variable is described by the graph of a function $\left\{s_{j}=N_{j}(f_{j})\right\}$ such that: 

(a) if one input to unit *j* is on (i.e., $f_{k}>\theta_{I}$ for some k≠j), then: 

(i) there is a monotone branch $N_{j}^{sil}$ of the $f_{j}$-nullcline,

(ii) $N_{j}^{sil}$ is defined on an interval $I_{j}^{sil}$ satisfying $f_{j}<\theta_{I}$ for all $f_{j}\in I_{j}^{sil}$,

(iii) $N_{j}^{sil}$ intersects the $s_{j}$-nullcline in a unique point $\left(f_{j}^{*},s_{j}^{*}\right)$, and

(iv) $(dN_{j}^{sil}(f_{j})/df)(ds_{j}/dt)>0$ when $ds_{j}/dt$ is evaluated along $N_{j}^{sil}$ with $f_{j}<f_{j}^{*}$;

(b) if no inputs to unit *j* are on, then: 

(i) there is a monotone branch $N_{j}^{act}$ of the $f_{j}$-nullcline,

(ii) $N_{j}^{act}$ is defined on an interval $I_{j}^{act}$ such that $\theta_{I}\in I_{j}^{act}$,

(iii) $N_{j}^{act}$ intersects the $s_{j}$-nullcline in a unique point $\left(f_{j}^{**},s_{j}^{**}\right)$ with $f_{j}^{**}<\theta_{I}$, and

(iv) $(dN_{j}^{act}(f_{j})/df)(ds_{j}/dt)<0$ when $ds_{j}/dt$ is evaluated along $N_{j}^{act}$ with $f_{j}>f_{j}^{**}$.

For system (1), each *v* plays the role of the fast variable *f* from (A1) while the other variable linked to *v* is the slow variable *s*. Since $S_{\infty }(v)$ is a Heaviside step function, (A2) holds for system (1), and the fact that all coupling is inhibitory, with a reversal potential less than the range of values traversed by each *v*, means that (A3) is satisfied as well. Assumption (A4), although more complicated than the others, is in fact fairly standard for typical planar neuronal models. This assumption holds, for example, if a unit’s *f*-nullcline is the graph of a cubic function for all levels of input; if in the presence of input, the nullcline’s left branch lies below $\theta_{I}$ and the unit has a critical point on this branch; and if in the absence of input, the nullcline’s right branch crosses through $\theta_{I}$, with a critical point on this branch having an *f*-coordinate less than $\theta_{I}$. It is easy to choose parameters for the $\left(v_{1},h\right)$ unit in system (1) that meet all of these criteria. The persistent sodium current renders the $v_{1}$-nullcline cubic, and we can choose $\theta_{I}$ and the parameters of $h_{\infty }$ to achieve the other desired properties, as we do throughout this article. The other two units in the system have monotone *v*-nullclines because each can be expressed as a graph (v,m(v)) where m(v) is the ratio of two linear functions of *v*. Certain choices of $\theta_{I}$ and parameters of $m_{\infty }$, such as those made in this article, ensure that (A4) holds for these units as well. We note that the assumptions made about the relations of the *f*-nullclines to $\theta_{I}$ can be weakened as long as $f_{j}=\theta_{I}$ is only achieved when the inputs to unit *j* are both off.

## Competing interests

The authors declare that they have no competing interests.

## Authors' contributions

JR and DT carried out the analysis, performed the numerical simulations, and wrote the paper.

## References

[B1] Rubin J Terman D Fiedler B Geometric singular perturbation analysis of neuronal dynamics Handbook of Dynamical Systems: Towards Applications 2 2002 Elsevier, Amsterdam

[B2] Ermentrout G Terman D Mathematical Foundations of Neuroscience 2010 Springer, New York

[B3] Lindsey B Rybak I Smith J Computational models and emergent properties of respiratory neural networks Compr Physiol 2012 2 1619 1670 10.1002/cphy.c110016 PMC3656479 23687564

[B4] Rybak I Abdala A Markin S Paton J Smith J Spatial organization and state-dependent mechanisms for respiratory rhythm and pattern generation Prog Brain Res 2007 165 201 220 17925248 10.1016/S0079-6123(06)65013-9 PMC2408750

[B5] Smith J Abdala A Koizumi H Rybak I Paton J Spatial and functional architecture of the mammalian brainstem respiratory network: a hierarchy of three oscillatory mechanisms J Neurophysiol 2007 98 3370 3387 10.1152/jn.00985.2007 17913982 PMC2225347

[B6] Rubin J Shevtsova NA Ermentrout GB Smith JC Rybak IA Multiple rhythmic states in a model of the respiratory central pattern generator J Neurophysiol 2009 101 2146 2165 10.1152/jn.90958.2008 19193773 PMC2695631

[B7] Molkov Y Abdala A Bacak B Smith J Rybak I Paton J Late-expiratory activity: emergence and interactions with respiratory CPG J Neurophysiol 2010 104 2713 2729 10.1152/jn.00334.2010 20884764 PMC2997033

[B8] Rubin J Bacak B Molkov Y Shevtsova N Rybak I Smith J Interacting oscillations in neural control of breathing: modeling and quantitative analysis J Comput Neurosci 2011 30 607 632 10.1007/s10827-010-0281-0 20927576 PMC3648224

[B9] Ben-Tal A Smith J A model for control of breathing in mammals: coupling neural dynamics to peripheral gas exchange and transport J Theor Biol 2008 251 480 497 10.1016/j.jtbi.2007.12.018 18262570 PMC2440661

[B10] Butera R Rinzel J Smith J Models of respiratory rhythm generation in the pre-Bötzinger complex. I. Bursting pacemaker neurons J Neurophysiol 1999 81 382 397 10400966 10.1152/jn.1999.82.1.382

[B11] DelNegro C Johnson S Butera R Smith J Models of respiratory rhythm generation in the pre-Bötzinger complex. III. Experimental tests of model predictions J Neurophysiol 2001 86 59 74 11431488 10.1152/jn.2001.86.1.59

[B12] Rybak I Shevtsova N Ptak K McCrimmon D Intrinsic bursting activity in the pre-Bötzinger complex: role of persistent sodium and potassium currents Biol Cybern 2004 90 59 74 10.1007/s00422-003-0447-1 14762725

[B13] Feldman J DelNegro C Looking for inspiration: new perspectives on respiratory rhythm Nat Rev, Neurosci 2006 7 232 242 10.1038/nrn1871 16495944 PMC2819067

[B14] Wang XJ Rinzel J Alternating and synchronous rhythms in reciprocally inhibitory model neurons Neural Comput 1992 4 84 97 10.1162/neco.1992.4.1.84

[B15] Golomb D Wang X Rinzel J Synchronization properties of spindle oscillations in a thalamic reticular nucleus model J Neurophysiol 1994 72 1109 1126 7807198 10.1152/jn.1994.72.3.1109

[B16] White J Chow C Ritt J Soto-Trevino C Kopell N Dynamics in heterogeneous, mutually inhibited neurons J Comput Neurosci 1998 5 5 16 10.1023/A:1008841325921 9580271

[B17] Whittington M Traub R Kopell N Ermentrout B Buhl E Inhibition-based rhythms: experimental and mathematical observations on network dynamics Int J Psychophysiol 2000 38 315 336 10.1016/S0167-8760(00)00173-2 11102670

[B18] Kopell N Börgers C Pervouchine D Malerba P Tort A Cutsuridis V, Graham B, Cobb S, Vida I Gamma and theta rhythms in biophysical models of hippocampal circuits Hippocampal Microcircuits Springer Series in Computational Neuroscience 5 2010 Springer, New York 423 457

[B19] Shilnikov A Belykh I Gordon R Polyrhythmic synchronization in bursting networking motifs Chaos 2008 18 3 Article ID 037120 10.1063/1.2959850 19045494

[B20] Matveev V Bose A Nadim F Capturing the bursting dynamics of a two-cell inhibitory network using a one-dimensional map J Comput Neurosci 2007 23 169 187 doi:10.1007/s10827-007-0026-x 10.1007/s10827-007-0026-x 17440801 PMC2606977

[B21] Chandrasekaran L Matveev V Bose A Multistability of clustered states in a globally inhibitory network Physica D 2009 238 253 263 10.1016/j.physd.2008.10.008

[B22] Wojcik J Clewley R Shilnikov A Order parameter for bursting polyrhythms in multifunctional central pattern generators Phys Rev E 2011 83 Article ID 056209 10.1103/PhysRevE.83.056209 21728632

[B23] Terman D Lee E Partial synchronization in a network of neural oscillators SIAM J Appl Math 1997 57 252 293 10.1137/S0036139994278925

[B24] Terman D Wang D Global competition and local cooperation in a network of neural oscillators Physica D 1995 81 148 176 10.1016/0167-2789(94)00205-5

[B25] Rubin J Terman D Analysis of clustered firing patterns in synaptically coupled networks of oscillators J Math Biol 2000 41 513 545 10.1007/s002850000065 11196583

[B26] Terman D Kopell N Bose A Dynamics of two mutually coupled inhibitory neurons Physica D 1998 117 241 275 10.1016/S0167-2789(97)00312-6

[B27] Rubin J Terman D Synchronized bursts and loss of synchrony among heterogeneous conditional oscillators SIAM J Appl Dyn Syst 2002 1 146 174 10.1137/S111111110240323X

[B28] Daun S Rubin JE Rybak IA Control of oscillation periods and phase durations in half-center central pattern generators: a comparative mechanistic analysis J Comput Neurosci 2009 27 3 36 10.1007/s10827-008-0124-4 19130197 PMC2844522

[B29] Tabak J O’Donovan M Rinzel J Differential control of active and silent phases in relaxation models of neuronal rhythms J Comput Neurosci 2006 21 307 328 doi:10.1007/s10827-006-8862-7 10.1007/s10827-006-8862-7 16896520

[B30] Butera R Rinzel J Smith J Models of respiratory rhythm generation in the pre-Bötzinger complex. II. Populations of coupled pacemaker neurons J Neurophysiol 1999 81 398 415 10400967 10.1152/jn.1999.82.1.398

